# Dual pathology: Everolimus‐induced microangiopathic haemolytic anaemia in a patient with myelodysplastic syndrome

**DOI:** 10.1002/jha2.416

**Published:** 2022-03-23

**Authors:** Ellen Nuttall Musson, Kate O'Connell, Neil Chauhan

**Affiliations:** ^1^ University College London London UK; ^2^ Royal Free Hospital London UK

1

A 72‐year‐old man with a background of myelodysplastic syndrome (MDS) and multiple endocrine neoplasia (type 1) presented with epistaxis.

He was clinically well with normal observations. His full blood count showed haemoglobin of 111 g/L, white cell count of 4.12 × 10^9^/L and platelets of 33 × 10^9^/L. His coagulation profile was normal and his renal function was at baseline. His platelet count 1 month previously had been 60 × 10^9^/L.

**FIGURE 1:**
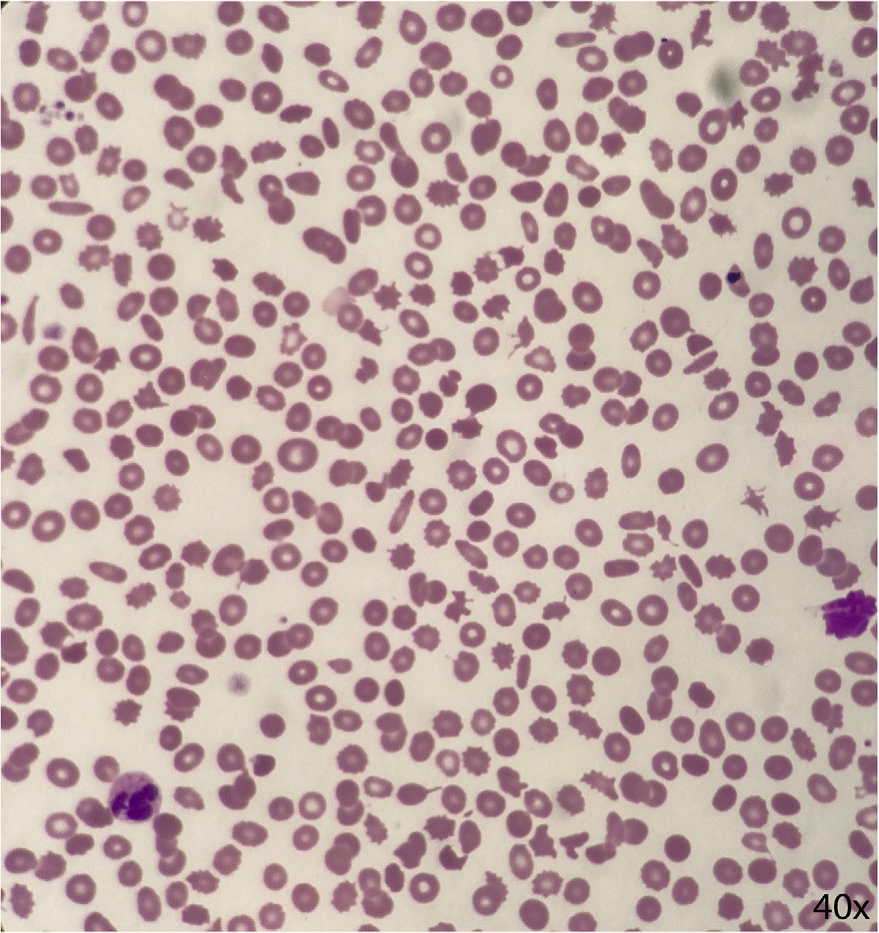
Peripheral blood film 10 days after initiation of everolimus therapy

His blood film (Figure 1) showed anisopoikilocytosis, platelet anisocytosis and hypolobated neutrophils (consistent with his known diagnosis of MDS), but also showed a new schistocytosis.

He had been commenced on everolimus therapy for progressive pancreatic neuroendocrine tumour with hepatic metastases 10 days previously. On admission, the everolimus was discontinued.

Subsequent blood tests showed a reticulocyte count of 17.6 × 10^9^/L, LDH 341 U/ml, haptogloin 1.81 g/L and an ADAMTS13 activity assay of 40 IU/dL. His haematinics were replete.

An element of everolimus‐induced thrombotic microangiopathy was suspected. Following cessation of the drug, his platelet count improved to baseline and a repeat blood film showed resolution of the schistocytosis, with persistent dysplastic changes.

Everolimus is an oral mTOR kinase inhibitor and is used in the treatment of breast cancer, renal cell carcinoma and neuroendocrine tumours. Everolimus can cause myelosuppression and is a rare but recognised cause of drug induced thrombotic microangiopathy. This image nicely illustrates some of the morphological features of MDS, the concerning finding of schistocytes on a blood film and serves as a helpful reminder of a rare cause of drug induced thrombotic microangiopathy.

